# Supplementary figures and images for: The flipped classroom is effective for medical students to improve deep tendon reflex examination skills: A mixed-method study
Source: PLoS One. 2022 Jun 17;17(6):e0270136. doi: 10.1371/journal.pone.0270136 (PMC9205501; doi:10.1371/journal.pone.0270136)

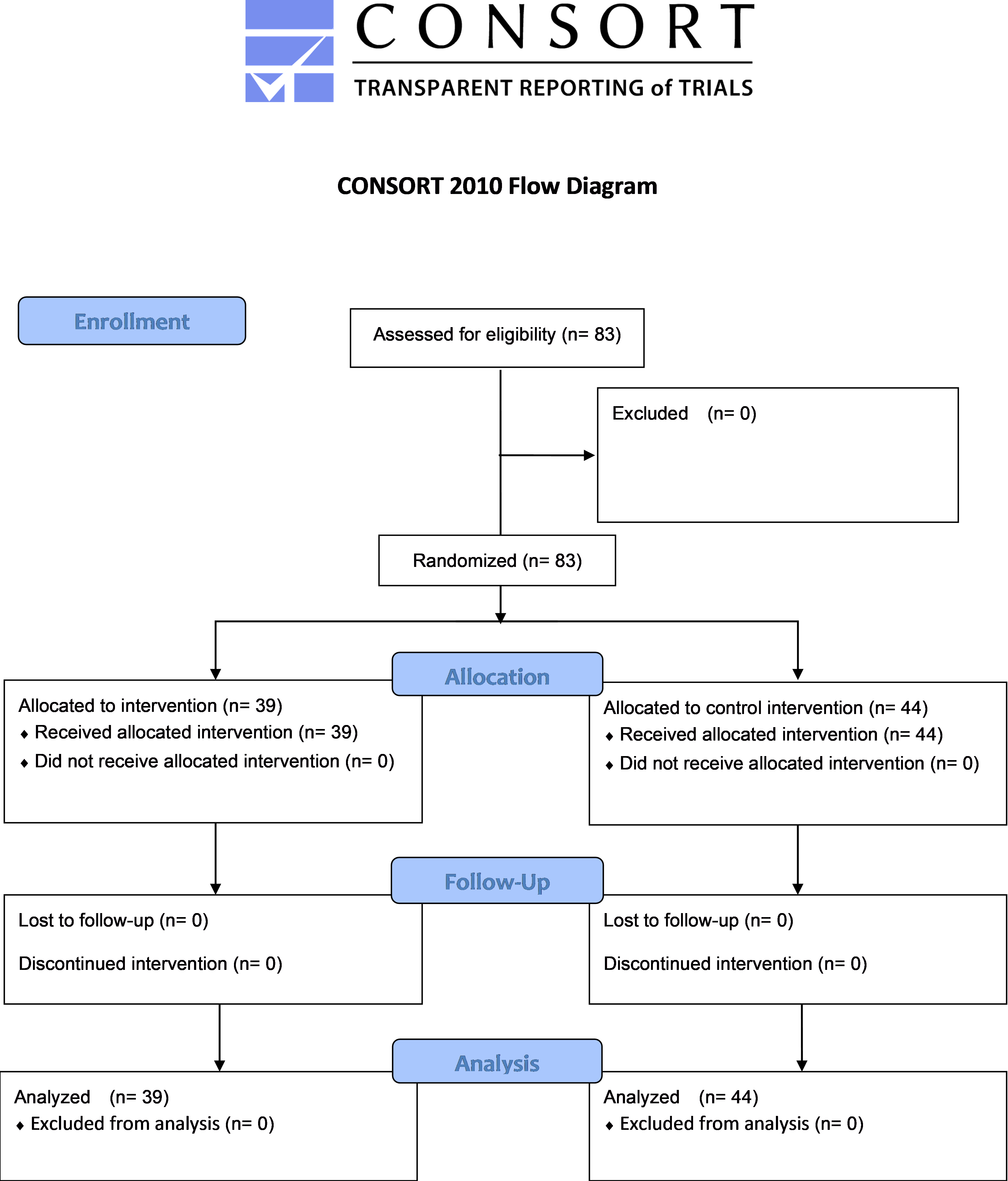

Supplement: S1 Fig — (TIF) [file pone.0270136.s001.tif]
