# Supplementary material for: The flipped classroom is effective for medical students to improve deep tendon reflex examination skills: A mixed-method study
Source: PLoS One. 2022 Jun 17;17(6):e0270136. doi: 10.1371/journal.pone.0270136 (PMC9205501; doi:10.1371/journal.pone.0270136)
Supplement: S1 Table — (PDF) [file pone.0270136.s002.pdf]

S1 Table. Interview guidelines

1. Introductory conversation	Thanks acknowledgment, purpose of this study, informed consent, permission to record on audiotape.
2. Lead the interview using the following questions (note: care should be taken to respect the flow of discussion)	Please introduce yourselves.<br>Think of the advantage of a flipped classroom.<br>Why do you feel it was an advantage?
3. Conclusion	Would anybody like to say anything else about this topic?
