# Supplementary material for: The flipped classroom is effective for medical students to improve deep tendon reflex examination skills: A mixed-method study
Source: PLoS One. 2022 Jun 17;17(6):e0270136. doi: 10.1371/journal.pone.0270136 (PMC9205501; doi:10.1371/journal.pone.0270136)
Supplement: Attachment — Submitted filename: PLOS One.docx [file pone.0270136.s004.docx]

**The flipped classroom is effective for medical students to improve deep tendon reflex 2 examination skills: A mixed-method study**

Reviewer	Ashfaq Akram

Line 86

The participants were allocated to the intervention or control groups.

**Comments:** *How the participants were divided into experimental and control group.* ***was it randomly or quota method?***

One group consisted of five to six students who practiced for two weeks in this department.

**Comment**: *This is not clear authors are talking about control / experimental group or Focus group.*

**Line 90**

**Participants**: A total of 83 medical students participated in the study. They were fifth-year students who participated in a clinical clerkship at the Department of General Medicine, Chiba University Hospital, from November 2018 to July 2019.

**Comment:** *How the study participants were recruited*; by using probably sampling or non-probably method. *How many students were in each group, this is not clear.*

**Line93**

One group consisted of five to six students who practiced for two weeks in this department. (*Confusing statement to the reader)*

Line 132

This sample size was more than 27, which is the required sample size for a two-tailed test of the difference in means between the two groups

**Comment**: *If the population size is 83 then with 95% confidence level and 3 margins of error, the sample size should be 68*

Line 137

The 15 groups were allocated to the intervention or control groups. The allocation was not blinded to the participants or the evaluators

**Comment***: How they are allocated the group this is not clear. If authors say, the allocation was not blinded to the participants, it could be bias that could alter the results.*

Looking at table 1

Self-confidence in DTR examinations (Pretest), mean (SD)*

Self-confidence in DTR examinations (Post-test), mean (SD)*

**Comment**: *Then authors must use paired t - test and they should add* ***t*** *value also in the table*

*Yes if they want to analyze experimental and control group after intervention ( e learning and face to face practice ) [not pre and post ]then they can use unpaired ( Independent ) t test .*

Line 151

In the intervention group, focus group interviews (FGI) [11-13] were conducted for a qualitative assessment.

Comment: *Why the focus group was chosen, they could choose randomly from both groups.*

*They can improve the manuscript by*

*Removing errors in sampling method, using tests to check normality of the data then if data is normally distributed then using paired t test ( Pre and Posttest for each group ) 2^nd^ option use unpaired t test but just post test data for each group*
