# Supplementary material for: The flipped classroom is effective for medical students to improve deep tendon reflex examination skills: A mixed-method study
Source: PLoS One. 2022 Jun 17;17(6):e0270136. doi: 10.1371/journal.pone.0270136 (PMC9205501; doi:10.1371/journal.pone.0270136)
Supplement: Attachment — Submitted filename: Response to Reviewers_20220515.docx [file pone.0270136.s005.docx]

Response to the Reviewers

May 15, 2022

**Title**: The flipped classroom is effective for medical students to improve deep tendon reflex examination skills: A mixed-method study
**Reference number**: PONE-D-21-37240

Dear Editors and Reviewers,

Thank you for your e-mail dated May 3, 2022 regarding our manuscript and the valuable comments of the associated editors and referees. I have attached our marked-up copy, the revised manuscript, and a point-by-point response to the associated editors’ and referees’ comments.

We hope that the revised manuscript incorporates appropriate revisions as responses to the comments. We believe it has been significantly improved over the initial submission. We trust that our manuscript is now eligible for publication in PLOS ONE. Thank you in advance for your kind consideration of our work.

Sincerely yours,
Shun Uchida, MD

Department of General Medicine, Chiba University Hospital
1-8-1, Inohana, Chuo-ku, Chiba-city, Chiba, Japan
Tel. +81-43-222-7171 (Ext. 6438); +81-43-224-4758 (Direct line)
Fax. +81-43-224-4758
E-Mail: shun.uchida@chiba-u.jp

# Responses to the comments by reviewer #1

## **NOTE**

We highlighted changes of significant issues in yellow (please see “Revised Manuscript with Track Changes”).

## Comment 1: Sample demographics should be discussed in the Methods (as should use of SD for statistics, when appropriate).

**Reply:**
We appreciate your suggestion. We have added the information on the sampling demographics, including age, sex, and completion of a clinical clerkship in neurology, in the Methods section.

**Change:**
Manuscript, Lines 108-110

“The participants' age, sex, and completion of a clinical clerkship in neurology, were collected as demographics.”

## Comment 2: The limitations of the study should also include the male sex bias in the sample demographics.

**Reply:**
Thank you for your comment. Although the distribution is typical for Japan, this may affect the results of this study. We have included this aspect in the Discussion section.

**Change:**
Manuscript, Lines 300-303

“In addition, there were more males than females among medical students at this institution (males = 80.7%, n=67 of 83), which may have affected the study results. This predominance of male medical students is characteristic of Japanese medical schools.”

We hope that the revised manuscript incorporates appropriate revisions as responses to the comments. We believe it has been significantly improved over the initial submission. We trust that our manuscript is now eligible for publication in PLOS ONE. Thank you in advance for your kind consideration of our work.

# Responses to the comments by reviewer #2

## **NOTE**

We highlighted changes of significant issues in yellow (please see “Revised Manuscript with Track Changes”).

## Comment 1: How the participants were divided into experimental and control group. was it randomly or quota method? (Line 86) Comment 6: How they are allocated the group this is not clear. If authors say, the allocation was not blinded to the participants, it could be bias that could alter the results. (Line 137)

**Reply:**
We appreciate your comment regarding our incorrect explanation. As stated in the “Randomization” section, we allocated groups, not individual participants, to the intervention and control groups. Therefore, as you have correctly pointed out, this is not the correct notation.
Hence, we have added this explanation in the “Randomization” section: “The groups were allocated through block randomization to the intervention and control groups.”
Kindly refer to our response to the Comment 3, mentioned below. This study was a part of a clinical clerkship education. The medical students were being rotated in groups assigned by the university before this study began. We allocated these groups to the intervention and control groups. This method could have led to a bias, as indicated by you. It should have been stated as a limitation.

**Change:**
Manuscript, Lines 94-96
“In the clinical clerkship, participants were being rotated in groups assigned by the university, before this study began. The groups of participants were allocated to the intervention or control groups.”

Manuscript, Lines 152-154
“The groups were allocated through block randomization using Microsoft Excel (Microsoft Co., Redmond, WA, USA) to the intervention and control groups.”

Manuscript, Lines 296-298
“In addition, since the university had assigned the groups before the start of this study, there might have been a bias in these assignments, which may have affected the study results.”

## Comment 2: This is not clear authors are talking about control / experimental group or Focus group. (Line 86)

**Reply:**
We apologize for our unclear explanation. We described focus group interviews separately.

**Change:**
Manuscript, Lines 97-98
 “In the intervention group, a focus group interview was planned for each rotating group as an additional qualitative assessment.”

## Comment 3: How the study participants were recruited; by using probably sampling or non-probably method. How many students were in each group, this is not clear. (Line 90)

**Reply:**
Thank you for highlighting this issue. As explained in the “Participants” section, the participants were artificially selected because this study was also a part of the clinical clerkship education in the department. This should have been specified in the “Participants” section.

**Change:**
Manuscript, Lines 103-106
“The participants were part of 124 fifth-year students and all students who were not yet practicing at the department. This study was also a part of the clinical clerkship education in the department; thus, the participants were not sampled randomly.”

## Comment 4: Confusing statement to the reader. (Line 93)

**Reply:**
As you pointed out, this statement was indeed confusing and contained explanations unrelated to the study; hence, we have corrected it.

**Change:**
Manuscript, Lines 106-107
“In this clinical clerkship, students were rotated in groups, each comprising five to six students.”

## Comment 5: If the population size is 83 then with 95% confidence level and 3 margins of error, the sample size should be 68. (Line 132)

**Reply:**
We appreciate your suggestion. As mentioned above, the participants were not a randomly selected sample from the population. Thus, the sample size was calculated by setting an effect size. However, the effect size was an inappropriate value. We recalculated the sample size with the effect size set to 0.8. We have added this information regarding the tools used in the calculations.

**Change:**
Manuscript, Lines 146-150
“Each group comprised more than 26 participants, which is the required sample size for a two-tailed test of the difference in means between the two groups, assuming a significance level of 0.05, a statistical power of 0.8, and an effect size of 0.8. The sample size was calculated using G*Power (Faul, Erdfelder, Lang, and Buchner, 2007) [11].”

## Comment 7: Then authors must use paired t-test and they should add t value also in the table. Yes if they want to analyze experimental and control group after intervention (e-learning and face to face practice) [not pre and post] then they can use unpaired (independent) t-test. (Line 137, Table 1)

**Reply:**
Thank you for this valuable suggestion. We analyzed using unpaired t-test because we planned to reveal the difference between the two groups. We did not analyze the change in self-confidence between pre- and post-test within the same group. As per your suggestion, we analyzed this using a paired t-test and found significant differences in both the intervention and control groups. We have added this result in the text and in a new table.

**Change:**
We have added Table 2. The previous Table 2, 3, and 4 are now Table 3, 4, and 5, respectively.

Abstract, Line 33-34
“Unpaired t-test was used to analyze the difference between the two groups on the results of the 5-point Likert scale and DOPS.”

Manuscript, Line 157-160
“All statistical analyses regarding the 5-point Likert scale and DOPS were performed using SPSS Statistics for Windows 26.0 (IBM Co., Armonk, NY, USA), setting the significance criterion at 0.05. An unpaired t-test was used to analyze the differences between the two groups. A paired t-test was used to analyze differences in pre-test and post-test within each group.”

Manuscript, Lines 203-205
 “Self-confidence in the post-test was significantly higher than that in the pre-test in both groups: t=7.7, P<0.001 in the intervention group and t=10.0, P<0.001 in the control group (Table 2).”

## Comment 8: Why the focus group was chosen, they could choose randomly from both groups. (Line 151)

**Reply:**
Thank you for your detailed observations. Indeed, conducting focus group interviews with both groups could have improved the study’s quality. When we designed the study, we had planned the focus group interviews to supplement the qualitative evaluation of the flipped classroom. We have added this background to the “Methods” section.

**Change:**
Manuscript, Line 169-170
“Because of the set of this theme, the FGI was conducted only in the intervention group.”

## Comment 9: They can improve the manuscript by removing errors in sampling method, using tests to check normality of the data then if data is normally distributed then using paired t test (Pre and Post-test for each group) 2nd option use unpaired t test but just post test data for each group.

**Reply:**
We appreciate your valuable suggestions. We have already explained the changes in previous responses to the related comments. Thanks to you, we were able to revise and improve our manuscript.

**Change:**

Abstract, Line 33-34
“Unpaired t-test was used to analyze the difference between the two groups on the results of the 5-point Likert scale and DOPS.”

Manuscript, Lines 146-150
“Each group comprised more than 26 participants, which is the required sample size for a two-tailed test of the difference in means between the two groups, assuming a significance level of 0.05, a statistical power of 0.8, and an effect size of 0.8. The sample size was calculated using G*Power (Faul, Erdfelder, Lang, and Buchner, 2007) [11].”

Manuscript, Lines 203-205
 “Self-confidence in the post-test was significantly higher than that in the pre-test in both groups: t=7.7, P<0.001 in the intervention group and t=10.0, P<0.001 in the control group (Table 2).”

We hope that the revised manuscript incorporates appropriate revisions as responses to the comments. We believe it has been significantly improved over the initial submission. We trust that our manuscript is now eligible for publication in PLOS ONE. Thank you in advance for your kind consideration of our work.
