# Supplementary material for: The flipped classroom is effective for medical students to improve deep tendon reflex examination skills: A mixed-method study
Source: PLoS One. 2022 Jun 17;17(6):e0270136. doi: 10.1371/journal.pone.0270136 (PMC9205501; doi:10.1371/journal.pone.0270136)
Supplement: Attachment — Submitted filename: Response to Reviewers_20220530.docx [file pone.0270136.s006.docx]

Response to the Reviewers

May 30, 2022

**Title**: The flipped classroom is effective for medical students to improve deep tendon reflex examination skills: A mixed-method study
**Reference number**: PONE-D-21-37240

Dear Editors and Reviewers,

Thank you for your e-mail dated May 27, 2022 regarding our manuscript and the valuable comments of the associated editors and referees. I have attached our marked-up copy, the revised manuscript, and a point-by-point response to the associated editors’ and referees’ comments.

We hope that the revised manuscript incorporates appropriate revisions as responses to the comments. We believe it has been significantly improved over the initial submission. We trust that our manuscript is now eligible for publication in PLOS ONE. Thank you in advance for your kind consideration of our work.

Sincerely yours,
Shun Uchida, MD

Department of General Medicine, Chiba University Hospital
1-8-1, Inohana, Chuo-ku, Chiba-city, Chiba, Japan
Tel. +81-43-222-7171 (Ext. 6438); +81-43-224-4758 (Direct line)
Fax. +81-43-224-4758
E-Mail: shun.uchida@chiba-u.jp

# Responses to the comments by reviewer #2

Note: We highlighted changes of significant issues in yellow (please see “Revised Manuscript with Track Changes”).

## **Comment 1**: There is no table for this statement authors should add Cohen's kappa values. (Line 136)

**Reply:**
Thanks for highlighting this issue. The inter-observer reliability could not be assessed by statistical method because research resources did not allow evaluation by multiple observers for a single participant. Instead, we attempted to clarify the DOPS evaluation criteria by faculty development to reduce inter-observer differences. As you pointed out, it should have been stated as a limitation.

**Changes:**
Manuscript, Lines 135-137
“The inter-observer reliability could not be assessed by statistical method because research resources did not allow evaluation by multiple observers for a single participant. Instead, the scaling criteria were defined through faculty development to improve inter-observer reliability.”

Manuscript, Lines 300-302
“the inter-observer reliability of DOPS-scored mastery of techniques was not statistically assessed. As noted above, multiple observers could not be provided for one examiner.”

## **Comment 2**: delete 15 groups it is confusing to the reader. Let ‘s suppose one group comprising 26 participants, then total should be 26 x 15 = 390 participants, it is incorrect because the authors said earlier 83 students of 5th year medical program …. (Line 146) **Comment 3**: The 15 groups were allocated to ……. medical students (n=83) were allocated into experimental (group A =43 or control groups (group B = 39) randomly (Line 152) **Comment 4**: authors could say experimental group n= 39 and control group n =44 (Lines 188-89)

**Reply:**
We apologize for our unclear explanation. As stated in the “Randomization” section, we allocated groups, not individual participants, to the intervention or control groups. We have revised the explanation about this allocation in the "Result" section more clearly.

**Changes:**
Manuscript, Lines 147-148
“A total of 83 medical students participated in the study. The required sample size was 26 in each group,”

Manuscript, Lines 188-190
“The 15 groups were allocated to the intervention group (7 groups) or the control group (8 groups). As a result, all participants (n=83) were assigned to the intervention group (n=39) or the control group (n=44).”

## **Comment 5**: (authors cannot analyse two groups with different intervention element) I think I suggested earlier they can choose Posttest values of (Table 1)

**Reply:**
We appreciate your essential remarks. This study compared two different classroom teaching approaches (flipped vs traditional). However, as you pointed out, pre-test self-confidence was inappropriate to compare the teaching approaches because it was made in the middle of the teaching intervention, as that would mean comparing different intervention elements. We corrected the manuscript to explain pre-test self-confidence was designed as an auxiliary assessment in the "Outcome measures" section.
Given the limited reliability of the DOPS, as pointed out in Comment #1, we no longer did not think each item in Table 1 was reliable enough to be highlighted. Therefore, we decided to delete the table.

**Changes:**

Manuscript, Lines 128-131
“After the procedural teaching, post-test self-confidence was scored on a 5-point Likert scale, and mastery of the techniques was evaluated using DOPS. As an adjunct, pre-test self-confidence before the procedural teaching was scored. That was scored after prior learning in the intervention group and before face-to-face teaching in the control group (Fig 1).”

Manuscript, Lines 210-212 (moving paragraph)
“Pre-test self-confidence in DTR examination (1=no confidence, 5=confidence) had mean (SD) values of 2.8 (0.8) and 2.3 (0.8) in the intervention and control groups, respectively—significantly higher in the intervention group (P=0.005).”

Table 1 (deleted)
Note: The previous Table 2, 3, 4, and 5 are now Table 1, 2, 3, and 4 respectively.

We hope that the revised manuscript incorporates appropriate revisions as responses to the comments. We believe it has been significantly improved over the initial submission. We trust that our manuscript is now eligible for publication in PLOS ONE. Thank you in advance for your kind consideration of our work.
